# A case of meningococcal meningitis with multiple cerebellar microbleeds detected by susceptibility-weighted imaging

**DOI:** 10.1186/s12880-015-0090-z

**Published:** 2015-10-22

**Authors:** Keita Miyazaki, Hidetada Fukushima, Youhei Kogeichi, Tomoo Watanabe, Kazunobu Norimoto, Toshiaki Taoka, Kazuo Okuchi

**Affiliations:** Department of Emergency and Critical Care Medicine, Nara Medical University, Shijo-cho, 840, Kashihara City, Nara Japan; Department of Neurosurgery, Nara Medical University, Shijo-cho, 840, Kashihara City, Nara Japan; Department of Neurosurgery, Nara Prefecture General Medical Center, Hiramatsu, 1-30-1, Nara City, Nara Japan; Department of Radiology, Nara Medical University, Shijo-cho, 840, Kashihara City, Nara Japan

**Keywords:** Bacterial meningitis, Magnetic resonance imaging, Susceptibility-weighted imaging, Bleeding

## Abstract

**Background:**

Bacterial meningitis is a fatal infectious disease of the central nervous system complicating intravascular involvements. Multiple microbleeds are rarely identified as complications because of the limited detection threshold of conventional imaging modalities. We report the first case of meningococcal meningitis with successful identification of multiple microbleeds in the cerebellum by susceptibility-weighted imaging.

**Case presentation:**

A 19-year-old Japanese female was brought to our emergency department because of fever and coma. A spinal tap was performed and turbid yellow fluid was collected. A diagnosis of bacterial meningitis was established and the patient was admitted to an intensive care unit. Dexamethasone and Antibiotics were administered and *Neisseria meningitides* was cultured from the spinal fluid. On day 10, postcontrast magnetic resonance imaging identified enhanced subarachnoid space in the cerebellum. Susceptibility-weighted imaging showed spotty low-intensity signals in the cerebellar tissue, indicating microbleeds. The patient made a full recovery from coma and was discharged without neurological sequelae on day 24.

**Conclusion:**

Meningococcal meningitis can cause multiple microbleeds in the cerebellum. In this report, we successfully identified microbleeds by susceptibility-weighed imaging. Using this imaging modality, further investigations will clarify its clinical incidence and significance.

## Background

Bacterial meningitis is a fatal infectious disease of the central nervous system with an annual incidence of 4–6 cases per 100,000 adults (patients older than 16 years of age). The most common pathogens are *Streptococcus pneumoniae*, *Neisseria meningitides*, and *Haemophilus influenzae*. Even with advanced medical care, mortality remains high between 10 and 40 % and severe neurological damage affects 30–52 % of survivors [1, 2].

Among the complications of bacterial meningitis, intracranial vascular involvement is well known, i.e., ischemic infarction, sinus thrombosis, and hemorrhagic complications [3]. According to both animal experiments and a human autopsy study, bacterial meningitis can also cause microbleeds [4–6]. However, microbleeds are not commonly identified in clinical practice because of the limited detection threshold of head computed tomography (CT) scans and conventional magnetic resonance imaging (MRI). Therefore, microbleeds, as a complication of bacterial meningitis, are rarely recognized.

In this report, we present a case of bacterial meningitis due to *N. meningitides* with successful identification of microbleeds in the cerebellum by susceptibility-weighted imaging (SWI), a sensitive method that can be used to detect microbleeds in brain tissue.

## Case presentation

A 19-year-old Japanese female without known medical history was transported to our emergency department because of coma. She complained of fever over 39 °C and severe occipital headache the night before her family found her unconscious. Physical examination revealed that her Glasgow coma scale was 6 (E1V1M4) with blood pressure of 128/72 mmHg, heart rate of 88 beats/min, and respiratory rate of 19 breaths/min. Both pupils were 3.5 mm with a sluggish light reflex. Neurological examination found no motor laterality or pathological reflex, except for nuchal rigidity. A head CT scan revealed no focal region, but diffuse swelling of the brain. Her blood tests showed elevated leukocytes (21,100 cells/μL) and C-reactive protein (20.6 mg/dL). A lumbar tap was performed and turbid yellow spinal fluid was collected. Spinal fluid examination revealed protein 394 mg/dl, glucose 0 mg/dl and a cell count of 10112/mm^3^ (96 % polymorphonuclear leukocytes), which confirmed the diagnosis of bacterial meningitis.

**Fig. 1:**
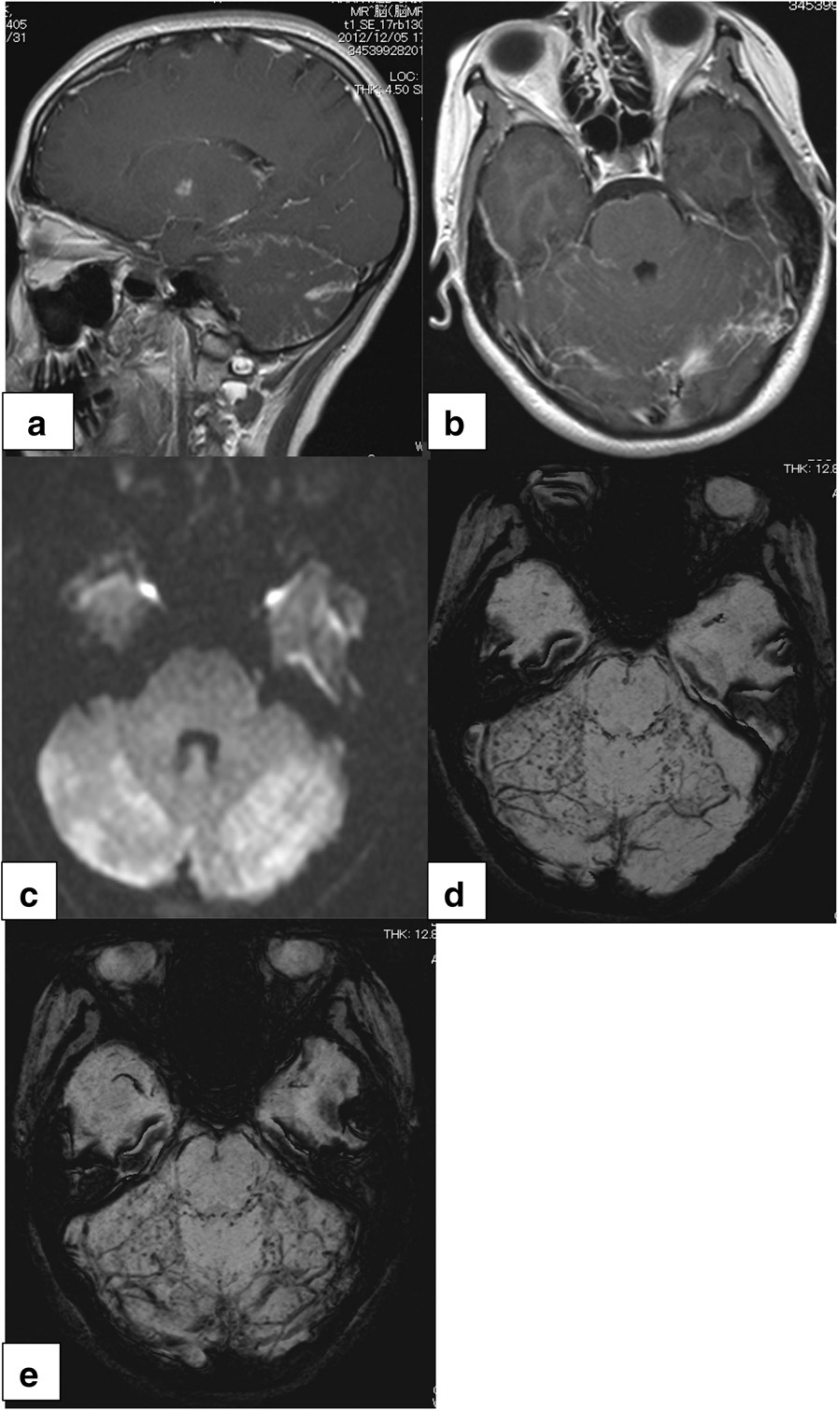
Brain magnetic resonance imaging of the patient. Postcontrast T1-weighted saggital and axial imaging (**a, b**) showed enhancement in subarachoid space of cerebellum, typically in the great horizontal fissure. Diffusion-weighted imaging (**c**) showed high-intensity area in the great horizontal fissure. Susceptibility-weighted imaging (**d**) showed nonlinear multiple low intensity spots. These multiple spots are not enhanced in axial postcontrast T1-weighted image (**b**). Follow up MRI on day 42 showed that the multiple low-intensity spots persisted, but were disappearing (**e**)

**Fig. 2:**
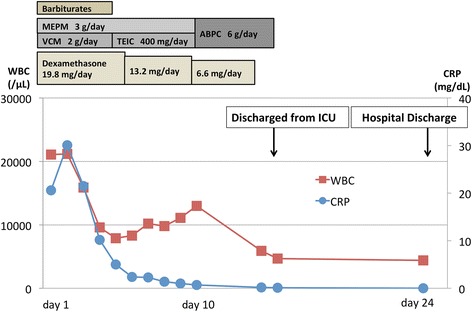
Clinical course of the case. The patient was managed successfully with antibiotics and dexamethasone and discharged on day 24. MEPM, meropenem; VCM, vancomycin; TEIC, teicoplanin; WBC, white blood cell; ICU, intensive care unit; ABPC, ampicilin

She was intubated and admitted to an intensive care unit. Due to poor study of the gram staining, meropenem and vancomycin were administered right after IV dexamethasone. On day 8, *N. meningitides* was cultured from the spinal fluid collected on admission and antibiotic therapy was changed to ampicillin (Fig. 2). Brain MRI study was performed on day 10 (Fig. 1). Postcontrast T1-weighted imaging showed enhanced subarachnoidal space of the cerebellum, typically along the great horizontal fissure (Fig. 1a and b). Additionally, Diffusion-weighted imaging (DWI) found high intensity area and SWI revealed multiple low-intensity spots in subarachoid space along the great horizontal fissure (Fig. 1c and d). Since these spotty low-intensity areas in the cerebellum located nonlinearly and there were no enhanced spots in the axial contrasted T1-weighted imaging, we confirmed that these low-intensity signals were not telangiectasia, but microbleeds. Antibiotic management was successful and her clinical symptoms improved. She was discharged on day 24 without neurological deficits. A follow-up brain MRI was performed on day 42. SWI showed the regions of multiple low-intensity spots in cerebellum persisted, but were disappearing (Fig. 1e). She had no neurological deficits and complained no symptoms on day 42.

We reported a case of meningitis due to *N. meningitides* in which the patient’s SWI findings indicated diffuse microbleeds in the cerebellum. Despite an extensive literature search, we could not find a report of bacterial meningitis with microbleeds in the cerebellum or cerebral cortex identified by SWI.

The most common MRI findings in bacterial meningitis are intraventricular signal alterations, meningeal enhancement, and intraparenchymatous signal changes [7]. In this case, postcontrast T1-weighted imaging revealed enhancement in subarachnoidal space of cerebellum, which is consistent with general MRI findings of bacterial meningitis. Interestingly, DWI showed high intensity areas and SWI revealed multiple low-intensity spots located with veins in subarachnoid space along the great horizontal fissure. On SWI, deoxyhemoglobin, hemorrhagic blood products, and methemoglobin of hemorrhagic lesions can be visualized as low-intensity signals [8]. With the findings of DWI and SWI, we speculated that a collection of purulent exudate in subarachnoid space caused microbleeds on the surface of cerebellum. Animal experiments and a recent autopsy study of bacterial meningitis reported that both macroscopic and microscopic hemorrhages can be observed [4, 6]. In a pneumococcal meningitis mouse model, microscopic hemorrhages can be found in both the subarachnoid space and cortex [4]. An autopsy study of pneumococcal meningitis by Vergouwen et al. [6] also reported that the majority of microbleeds were located in the cortex and subcortex with no evidence of infarction. The mechanism of microbleeds is considered as pyogenic vasculitis following vascular wall injury or extravasation of red blood cells from congested small veins caused by severe inflammation [6]. Microbleeds in meningococcal meningitis, however, has not been observed in human studies.

Other pathophysiology which can cause microbleeds detected by SWI are hypertsensive vasculopathy or amyloid angiopathy [9]. This patient, however, had no history indicating these pathophysiologies.

The clinical significance of microbleeds in bacterial meningitis, however, is not well documented. In this current case report, the cerebellar microbleeds did not seem to cause any neurological deterioration. Further studies are needed to investigate the pathogenesis of microbleeds and their clinical significance.

## Conclusions

Meningococcal meningitis can cause multiple microbleeds in the cerebellum. In this report, we successfully identified microbleeds by susceptibility-weighed imaging. Using this imaging modality, further investigations will clarify its clinical incidence and significance.

## Consent

Written informed consent was obtained from the patient for publication of this case report and any accompanying images. A copy of the written consent is available for review by the Editor of this journal.

## Abbreviations

CT: Computed tomography
MRI: Magnetic resonance imaging
DWI: Diffusion-weighted imaging
SWI: Susceptibility-weighted imaging
